# Molecular Characterization and Functional Analysis of the *Hb-hsp*90-1 Gene in Relation to Temperature Changes in *Heterorhabditis bacteriophora*

**DOI:** 10.3389/fphys.2021.615653

**Published:** 2021-02-23

**Authors:** Elena Fanelli, Alberto Troccoli, Eustachio Tarasco, Francesca De Luca

**Affiliations:** ^1^Institute for Sustainable Plant Protection-CNR, Bari, Italy; ^2^Section of Entomology and Zoology, Department of Soil, Plant and Food Sciences, University of Bari “A. Moro”, Bari, Italy

**Keywords:** acclimation, EPN, gene duplication, heat shock protein, thermotolerance, silencing

## Abstract

Understanding how entomopathogenic nematodes respond to temperature changes and have adapted to the local environment is crucial to improve their potential as biocontrol agents. In order to improve understanding of *Heterorhabditis bacteriophora*’s potential adaptability to future climate changes, full-length cDNA and the corresponding gene of heat shock protein 90 (Hsp90) were isolated and fully characterized. The reproductive potential of the Apulian strain of *H. bacteriophora* increased when the temperature rose from 23 to 30°C, but no reproduction was found at 12°C. Expression analyses revealed that *Hb-hsp*90-1 was differentially expressed in Infective Juveniles (IJs) and adults (hermaphrodites, females and males). Up-regulation of *Hb-hsp*90-1 was higher during the recovery process in *Galleria mellonella* larvae than adults, thus confirming the protective role of *Hb-hsp*90-1 in coping with the host environment. Silencing of *Hb-hsp*90-1 resulted in a significant reduction (76%) in the expression level. Silenced IJs took longer than untreated nematodes to infect *G. mellonella*, showing that *Hb-hsp*90-1 could be also involved in chemosensation. Furthermore, the number of adults and IJs recovered from *G. mellonella* infected with silenced nematodes and incubated at 30°C was higher than that obtained from *G. mellonella* infected with untreated nematodes. These data confirm the crucial role of *Hb-hsp*90-1 allowing acclimation to increased temperatures and modulation of the recovery process.

## Introduction

Entomopathogenic nematodes (EPNs) of Steinernematidae and Heterorhabditidae families are obligate parasites of a wide range of insect pests (Lacey et al., 2015); they are used as biocontrol agents, mostly for soil-dwelling insects. Like most plant parasitic nematodes, EPNs infect host insects as infective juveniles (IJs), which are developmentally arrested third-stage larvae. IJs are long-lived, non-feeding, stress-resistant, and able to locate specific insects using chemosensory cues (Chaisson and Hallem, 2012; Rasmann et al., 2012). IJs carry symbiotic bacteria in their intestines: *Photorhabdu*s spp. are associated with heterorhabditids and *Xenorhabdus* spp. with steinernematids. Inside a suitable host, IJs release these symbiotic bacteria into the insect’s hemocoel, killing the host in 24–48 h and thus providing nutrients for the nematodes (Lewis and Clarke, 2012).

Their pathogenicity in the field depends on several biotic and abiotic conditions, including temperature, humidity, soil type, application timing, and genetic traits (Grewal et al., 2006). Moreover, the effect of soil temperature on infectivity, survival and host-seeking behavior of EPNs is well known to vary among species and strains (Mukuka et al., 2010b; Lee et al., 2016). However, nematodes are also able to cope with the effects of harmful temperatures by using mechanisms such as heat shock response, developmental arrest and behavioral strategy (Mukuka et al., 2010a, b). Heat shock response is a highly conserved strategy among nematodes, but closely related species and strains of EPNs can differ greatly in their tolerance to higher temperatures (Mukuka et al., 2010a; Tarasco et al., 2015). This suggests that temperature tolerance can evolve rapidly. The way a species responds to heat stress affects not only its immediate tolerance level, but can also affect its long-term potential to increase tolerance. Hashmi et al. (1997) reported a correlation between the geographical origin of *Heterorhabditis* spp. and polymorphism of the *hsp*70A gene, highlighting that heat shock response to increasing temperature can affect the tolerance of *Heterorhabditis* strains.

Temperature is particularly crucial to organismal fitness and to behavior. To date very little is known about the heat shock genes involved in the response of *Heterorhabditis bacteriophora* to temperature change.

Hsp90s are involved in protein folding, protecting the proteome from misfolding and aggregation. All organisms except Archaea (Gupta, 1995; Chen et al., 2003) possess them, and they are highly conserved. Hsp90s are essential for viability under all conditions in eukaryotes, they are expressed in response to adverse environmental or chemical stresses and pathophysiological situations, and play an important role in cell survival (Pantzartzi et al., 2013). The Hsp90 gene family has undergone major duplication events leading to isoforms with cellular compartmentalization. In all vertebrates studied to date there are two known cytoplasmic isoforms which derived from duplication events. However, additional duplication events have occurred, thus increasing the number of total gene copies observed in vertebrates (Hoter et al., 2018). A total of 13 cytoplasmic genes have been identified in humans, 9 of which are pseudogenes, whereas the number of cytoplasmic gene copies in invertebrates is not uniform. To date, a single gene encoding for Hsp90 has been reported in nematodes and in *Drosophila* (Konstantopoulou and Scouras, 1998; Inoue et al., 2003; De Luca et al., 2009). Recently, two gene copies for *hsp*90 have been found in plant parasitic nematodes, differing each other in nucleotide sequence and intron–exon structure (Fanelli et al., 2018).

The present study reports on the isolation, gene organization and functional characterization of the *Hb*-*hsp*90-1 gene in *H. bacteriophora*. This research also investigated the ability of *H. bacteriophora* to adapt to future temperature changes in long-term experiments using decreased and increased temperatures and testing how the entomopathogenic nematode responds to increased temperature during the recovery process at the molecular level. Our functional characterization of the *Hb*-*hsp*90-1 gene demonstrated its evolutionary adaptive response to soil and insect environments, including an expanded gene family for *hsp*90 and expression profiles within increased temperature.

## Materials and Methods

### EPN Culture

Apulian EPN strain *H. bacteriophora* (LU1), kindly provided by Prof. Tarasco (Bari, Italy) was maintained under laboratory conditions using last instar-larvae of *G. mellonella* (L.) as the host (Woodring and Kaya, 1988). The infected *G. mellonella* cadavers were placed on White traps in order to obtain suspension of IJs (White, 1927). Harvested IJs were washed with distilled water three times, and stored at 15°C before use.

### DNA and RNA Extraction

Total genomic DNA and total RNA were extracted from 2,000 frozen IJ stages which were ground in liquid nitrogen using a pestle and mortar. Total genomic DNA and RNA were extracted from the resulting powder using an AllPrep DNA/RNA kit (QIAGEN) for simultaneous purification of nucleic acids according to the manufacturer’s instructions.

### Isolation of *Hb*-*hsp*90-1 Gene

Amplification of genomic DNA fragments was conducted on 50 ng of *H. bacteriophora* DNA with degenerate primers: U831 (5′-AAYAARACMAAGCCNTYTGGAC-3′) and L1110 (5′–TCRCARTTVTCCATGATRAAVAC–3′) (Skantar and Carta, 2004). PCR was performed with Taq DNA polymerase (Promega) as follows: initial denaturation at 94°C for 2 min, followed by 45 cycles of denaturation at 94°C for 20 s, annealing at 65°C for 5 s, at 60°C for 5 s, at 55°C for 5 s, 50°C for 5 s and extension at 68°C for 1 min with a final step at 68°C for 15 min. PCR product was extracted from the gel using the protocol provided by the manufacturer (NucleoSpin Gel and PCR Clean-up, Machery Nagel, Germany) and cloned into pGem T-easy vector System II (Promega). Plasmid inserts were sequenced and analyzed. The same procedure was used to amplify the partial *Hb-hsp*90 gene in individual nematodes (De Luca et al., 2004).

### 3′/5′ RACE

The 3′/5′ RACE of *Hb-hsp*90-1 was conducted on 1 μg of *H. bacteriophora* total RNA using SMART SMARTer RACE 5′/3′ Clontech, following the manufacturer’s protocol. The 3′ end of *Hb-hsp*90-1 was amplified using the gene specific primer EPNhsp3UTR1 (GACGACATCTCAAACGAGGA) and a long universal primer UPM (CTAATACGACTCAC TATAGGGCAAGCAGTGGTATCAACGCAGAGT).

PCR was performed as follows: 30 cycles at 94°C for 30 s, 64°C for 30 s, and 72°C for 90 s; and a final extension step at 72°C for 7 min. Using the short universal primer UPS (CTAATACGACTCACTATAGGGC) and either EPNhsp3UTR2 (GCCGTGAAGCACTTCTCCGT) or EPNhsp3UTR3 (GAAGGGCAGCTTGAGTTCCG) specific primers, nested PCR generated a band that was cloned and sequenced. The 5′ end of *Hb-hsp*90-1 was generated using the gene specific primer EPNhsp5UTR3 (CGGAACTCAAGCTGCCCTTC) and a long Universal Primer Mix UPM. PCR was performed as follows: 30 cycles at 94°C for 30 s, 62°C for 30 s, and 72°C for 2 min; and a final extension step at 72°C for 7 min. Using the short universal primer UPS and gene specific primer EPNhsp5UTR1 (TCCTCGTTTGAGATGTCGTC), nested PCR generated a band that was cloned and sequenced.

Sequences obtained with 3′/5′ RACE were used to draw primers 5′UTRhspHbFor (GCGGAACCAAGGATGTCTGACG) and 3′UTRhspHbrev (TGTGATCTCTGCGTCATCTCG), which were used to amplify the entire gene and to verify the continuity of the sequence on cDNA.

### Expression Pattern Analysis of *Hb*-*hsp*90-1

Total RNA was extracted from 1000 IJs and 500 adults of *H. bacteriophora* using RNeasy Tissue Mini Kit following the manufacturer’s instructions (Qiagen), and then treated with RNase-free DNase I set (Qiagen) to eliminate any contaminating genomic DNA. First-strand cDNA was synthesized from 50 ng of total RNA using a QuantiTect Reverse transcription kit (Qiagen) following the manufacturer’s instructions.

The ΔΔCt method was used to calculate relative expression among life stages and a portion of the 18S rRNA gene was used as endogenous control to normalize the transcription levels.

Real-time PCR was performed in 20 μl volumes containing 4 ng of cDNA, 10 μl 2 x Fast Start SYBR Green master mix (Roche) and 8 pmol of each specific primer.

The gene-specific primers used were hspHbfor3 (GAGCCTCAGTCACATGCTAC)/3′UTRhspHbrev (TGTGAT CTCTGCGTCATCTCG). The thermal profile for real-time PCR was 10 min at 95°C followed by 40 cycles at 95°C for 30 s, at 58°C for 30 s, and 72°C for 40 s. Dissociation curve analysis of amplification products was performed at the end of each PCR to confirm that only one PCR product was amplified and detected: 1 min at 95°C, 30 s at 55°C and 30 s at 95°C. The real-time experiments were conducted on a Stratagene thermal cycler and fluorescent real-time PCR data were analyzed using MX3000P software.

### Determination of Temperature Tolerance

Procedures described by Mukuka et al. (2010b), were followed in order to estimate the range of temperatures at which the Apulian *H. bacteriophora* (LU1) can infect last-instar larvae of *G. mellonella*. Twenty replicates of last-instar larvae of *G. mellonella* were used for each temperature (12, 23, and 30°C) and were infected with 600 IJs of *H. bacteriophora* (30 IJs for each replicate) and were placed in three separate incubators; 23°C was considered as the control temperature. Fifty adults (hermaphrodites, females and males) and 100 IJs which emerged from dissected *G. mellonella* cadavers, respectively, at five and nine days after infection, were collected, washed with distilled water, frozen in liquid nitrogen and stored at –80°C before use.

In parallel, 50 adult hermaphrodites and 100 IJs, which emerged from *G. mellonella* cadavers were placed separately in water suspension, for 6 h at 23, 12, and 30°C. After incubation in aqueous solution, the nematode samples were frozen in liquid nitrogen and stored at −80°C before use.

Total RNA and expression profiles of *Hb-hsp*90-1 were performed as described in section “3′/5′ RACE.”

### Double Stranded RNA (dsRNA) Preparation for *Hb*-*hsp*90-1

Templates for dsRNA were made by PCR on cDNA generated from RNA extracted from the aqueous suspension of *H. bacteriophora* IJs using a RNeasy kit (QIAGEN) according to the manufacturer’s instructions.

Two separate PCR reactions were performed, in which a fragment of *Hb*-*hsp*-90-1 gene was amplified with a T7 promoter sequence incorporated at the 5′ end of either the sense or antisense strand.

The *Hb*-*hsp*-90-1 primer sequences used for the reactions were T7hspHbfor3 (TAATACGACTCACTATAGGGGAGCCT CAGTCACATGCTAC) and 3′UTRhspHbrev (TGTGATC TCTGCGTCATCTCG) in the first reaction, and hspHbfor3 (GAGCCTCAGTCACATGCTAC) and T73′UTRhspHbrev (TAATACGACTCACTATAGGGTGTGATCTCTGCGTCATCT CG) in the second reaction.

PCR products were cleaned using a PCR purification kit (QIAGEN) and 1 μg of each PCR product was used for *in vitro* transcription with a Megascript kit (Ambion) according to the manufacturer’s instructions. The RNA generated in the two reactions was annealed to generate dsRNA. DNA and single-stranded RNA were removed by nuclease digestion (Megascript kit; Ambion). dsRNA was purified using filter cartridges (Ambion) and eluted in 100 μl of elution solution (Ambion). The dsRNA was verified by 1% agarose gel electrophoresis and quantified using a NanoDrop spectrophotometer. Three hundred *H. bacteriophora* IJs were collected and soaked in 40 μl *Hb*-*Hsp-*90-1dsRNA solution (1 μg/μl) for 24, 48, and 72 h, while controls were incubated in elution buffer. Treated and control nematodes were cleaned three times with DEPC-treated water and total RNA was then extracted. qPCR was used to analyze transcript suppression after RNAi treatment. All experiments were performed three times.

A specific portion of 18S rRNA gene was used as the endogenous control. Real-time PCR was performed in 20 μl volumes containing 6 ng of cDNA, 10 μl of 2 × Fast Start SYBR Green master mix (Roche) and 8 pmol of each specific primer.

Gene-specific primers used to analyze *Hb-hsp*90-1 transcript suppression were hspHbfor (GGCTCAAGCATTACG TGATTCG) and qPCR hspHbrev (TGAGGCTCCTCCAGA GTGA). PCR was as follows: 10 min at 95°C followed by 40 cycles at 95°C for 30 s, 58°C for 30 s, and 72°C for 40 s. Dissociation curve analysis of amplification products was also performed.

### RNAi by Soaking

Six hundred IJs were kept in 30 μl of *Hb-hsp*90-1 dsRNA (1 μg/μl)-treated nematodes and in 30 μl of soaking buffer-untreated nematodes for 48 h. Three *G. mellonella* larvae were transferred into one sterile 50 mm Petri dishes containing three Whatmann paper disks. Hundred treated and untreated IJs, were used to infect 3 *G. mellonella* in each Petri dish (approximately 30 IJs per *G. melonella*) at two different temperatures 23 and 30°C. The bioassay was repeated three times for each temperature and treatment.

Mortality was recorded after 24 and 48 h for each treatment and temperature. For each treatment, three dead larvae were dissected after 5 days and three dead larvae after 10 days and three dead larvae were left in White traps to recover the progeny. Adult, hermaphrodites and IJs were recovered and counted at 5 and 10 days.

### *Hb-hsp*90*-*1 Silencing Effect on the Motility of IJs

The effect of *Hb*-*hsp*90-1 silencing on nematode motility was determined. Thirty treated and untreated nematodes *H. bacteriophora* IJs were placed in the center of a 90 mm plate containing 3 mm water agar (1% w/v) in a 30 μl aliquot of ds buffer. For each plate one *G. mellonella* larva was placed at a distance of 40 mm from the nematodes and three replicates were conducted for each treatment. Petri dishes were incubated at 23°C in darkness and checked after 4 h for IJs movement. After 4 h evaporation of the ds buffer allowed the IJs to begin movement over the agar surface. RNAi treated and untreated nematode were observed under microscope in the release point. After 24 h, untreated IJs were not present in the release point, whereas several treated IJs were still found in the release point. After 48 h all treated IJs were not found in the release point.

### Data Analysis

Data were pooled and analyzed using a general linear model procedure (ANOVA) and significant differences between means were separated using Tukey’s HSD test (STATISTIX 9.0, 2008). All comparisons were made at 0.05 level of significance.

## Results

### Molecular Characterization of *Hb*-*hsp*90-1

A PCR reaction on *H. bacteriophora* genomic DNA using degenerate primers produced two fragments of 255 and 366 bp. A BLASTX search of these sequences confirmed that both corresponded to heat shock protein90. The 366 bp sequence differed from the 255bp fragment that it had two introns, which were 58 and 54 bp in length, respectively. No introns were present in the 255 bp fragment. Sequence analyses of six clones containing the 255 bp fragments revealed the presence of three different homologous sequences to *hsp*90 without introns.

The two bands of 255 and 366 bp were also amplified in individual specimens of *H. bacteriophora*. The 366 bp sequence was identical to that obtained on gDNA and was named *Hb-hsp*90-1, while three clones containing the 255 bp fragments were intronless and identical to those already obtained on the gDNA. The intronless isoforms were named *Hb-hsp*90-2, *Hb-hsp*90-3, *Hb-hsp*90-4 and *Hb-hsp*90-5. The intronless *Hb-hsp*90-2 showed a 100% identity with *Hb-hsp*90-1, while the remaining isoforms without introns showed a 75% similarity to each other (64/85 identities) and 91% positive (78/85) at the amino acid level. The intron-rich isoform *Hb-hsp*90-1 showed a 74–75% similarity to the other three intron less isoforms. Since all independent clones corresponding to the four partial *Hb-hsp*90 genes isolated in *H. bacteriophora* were correctly translated, they cannot be attributed to sequencing errors. All eight intron-rich clones sequenced showed six nucleotide substitutions some of these polymorphisms were present in more than one clone and they do not change the amino acid sequence. Pairwise comparisons between the different clones of intronless *Hb-hsp*90 isoforms revealed a 98–99% similarity within each group, while the *Hb-hsp*90-3 isoform showed a 74 and 75% similarity with *Hb-hsp*90-4 and *Hb-hsp*90-5, respectively. Pairwise comparison between *Hb-hsp*90-4 and *Hb-hsp*90-5 isoforms showed 89% similarity. These partial intronless isoforms showed 33–36 nucleotide differences compared with the corresponding region of the *Hb-hsp*90-1 gene. All intron losses in our data set are exact.

The 366 bp intron rich fragment was fully characterized and the complete *Hb-hsp*90-1 gene and the corresponding cDNA were determined.

3′/5′ RACE experiments on the 366 bp produced the full-length cDNA of the *Hb*-*hsp*90-1. The full-length *Hb*-*hsp*90-1 cDNA was 2318 bp long, containing an open reading frame of 2139 bp, a 42 bp 5′ UTR and a 137 bp 3′ UTR containing the polyadenylation signal (AATAAA).

**FIGURE 1:**
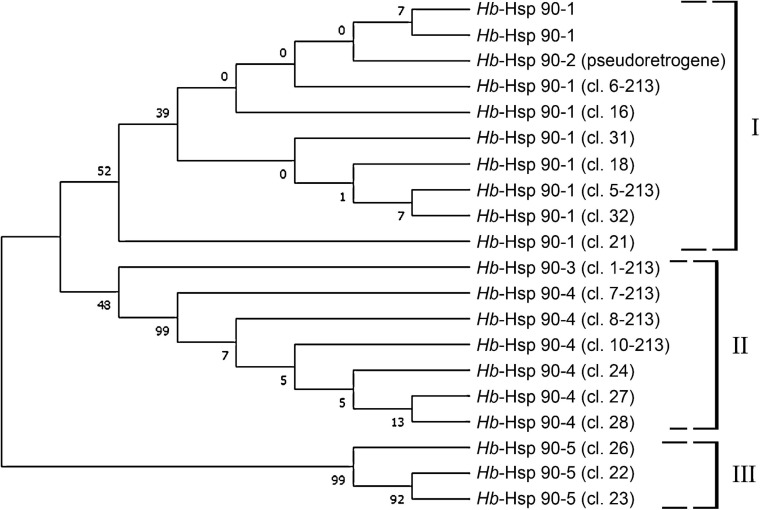
Phylogenetic analysis of the deduced amino acid sequences of Hsp90 isoforms from *Heterorhabditis bacteriophora* reveals the presence of distinct groupings and the duplication of the retropseudogene Hsp90-2 occurred relatively recently in *Heterorhabditis bacteriophora.*

Amplification of *H. bacteriophora* gDNA using a primer on 5′UTR region in combination with a primer used for a 5′ end RACE gave two products: a 1250 bp fragment, corresponding to the gene region encompassing exon and intron sequences, and a 950 bp intronless fragment, *Hb-hsp*90-2, identical to the *Hb-hsp*90-1 cDNA. Amplification of *H. bacteriophora* gDNA, using a primer on the 3′UTR region in combination with a primer used for a 3′end RACE, produced only one fragment that shows the presence of introns. Amplification of *H. bacteriophora* gDNA, using primers located in the UTR regions of the cDNA produced only one fragment of 2789 bp for *Hb*-*hsp*90-1. Thus, *Hb-hsp*90-2 can be designed as a retrogene showing 3–9 nucleotide differences from *Hb-hsp*90-1 cDNA and indicating a recent duplication. Phylogenetic analyses of all *Hb-hsp*90 sequences obtained clustered in three different groups with high support (Figure 1): Group I included all *Hb-hsp*90 sequences with introns and the retrogene, while Group II and III were all intronless sequences.

**FIGURE 2:**
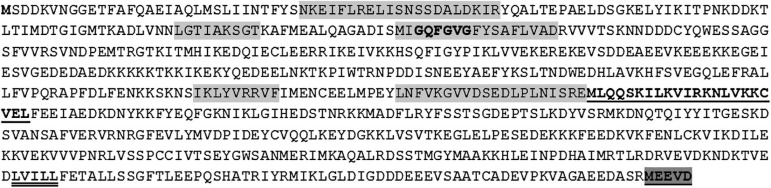
Deduced amino acid sequence of the *Hb*-*Hsp*90-1 of *Heterorhabditis bacteriophora*. The five Hsp90 signature sequences are shaded in light gray. GxxGxG motif is indicated in bold. The consensus leucine zipper sequence is double underlined. The consensus LxxLL sequence is indicated in bold underlined. The cytoplasmic Hsp90 sequence motif (MEEVD) is underlined and shaded in dark gray.

The *Hb-hsp*90-1 ORF encoded a deduced protein of 712 amino acids showing the five typical features (Figure 2) common to all eukaryotic Hsp90 proteins, and the GxxGxG motif, consensus leucine zipper sequence, consensus LXXLL sequence and cytoplasmic Hsp90 sequence motif (MEEVD) are also present. The calculated molecular weight is 81,67 kDa and a theoretical pI is 4.87. A BLASTP search of the GenBank database using the *Hb*-Hsp90-1 amino acid sequence revealed that the best matches were to other nematodes including *Ancylostoma ceylanicum* (98% identical/96% similar/100% coverage), *Ancylostoma duodenale* (93%/96%/100%), *Haemonchus contortu*s (93%/96%/100%), *Angiostrongylus cantonensis* (93%/96%/100% coverage), *Necator americanus* (93%/97%/100%), *Caenorhabditis brenneri* (89%/95%/100%), *Caenorhabditis elegans* (87%/94%/100%), *Anisakis brevispiculata* (84%/92%/100%), *Brugia pahangi* (84%/92%/100% *coverage*), *Bursaphelenchus xylophilus* (84%/92%/100%), *Bursaphelenchus doui* (83%/92%/100%), *Steinernema carpocapsae* (83%/92%/100%), *Steinernema feltiae* (81%/91%/100%), *Ditylenchus destructor* (81%/91%/100%), *Heterodera glycines* (80%/90%/100%), *Meloidogyne incognita* (79%/89%/100%), and *Meloidogyne artiellia* (78%/89%/100%). The newly obtained sequences were submitted to GenBank with the following accession numbers: LR890107–LR890114 for *Hb-hsp*90-1 sequences; LR890106 for mRNA *Hb-hsp*90-1; LR890105 for *Hb-hsp*90-2; LR890115–LR890117 for *Hb-hsp*90-3; LR890118–LR890120 for *Hb-hsp*90-3; LR890121–LR890123 for *Hb-hsp*90-5.

### Gene Structure

The exon–intron boundaries of the genomic sequence of *Hb*-*hsp*90-1 gene were determined by aligning the genomic with the corresponding cDNA sequence which revealed 12 exons and 11 introns. The introns ranged in size from 54 to 70 bp and intron-exon junctions GT and AG were conserved.

**FIGURE 3:**
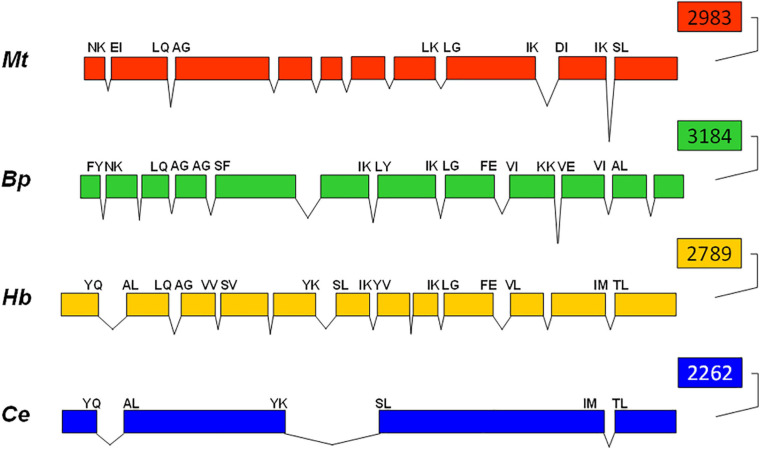
Comparison of intron-exon structure of *Hb*-*hsp*90-1 of *Heterorhabditis bacteriophora* compared with those of *Meloidogyne artiellia*, *Brugia pahangi*, and *Caenorhabditis elegans*. Exons are shown as boxes and introns as V-shaped lines. The amino acid at intron junctions indicated conserved positions between nematodes.

Comparison of the complete *hsp*90 gene sequences from the free-living nematode *C. elegans* (Birnby et al., 2000), parasitic nematodes *B. pahangi* (Thompson et al., 2001) and *M. artiellia* (De Luca et al., 2009) and entomopathogenic nematode *H. bacteriophora* revealed that the number of introns is variable ranging from three introns in the free-living nematode to 9 in *M. artiellia* and 11 in *H. bacteriophora* and *B. pahangi* (Figure 3). Only the position of the last intron is conserved among the four nematodes. However, four more intron positions are conserved between *Hb*-*hs*p90-1 and *Bp*-*hsp*83 genes: the second, sixth, eighth, and ninth introns from *H. bacteriophora* are coincident with the third, sixth, seventh, and eighth in *B. pahangi* (Figure 3). Two more intron positions are conserved between *Hb*-*hsp*90-1 and *Mt*-*hsp*90 genes: the second and eighth introns from *H. bacteriophora* coincide with the second and eighth in *M. artiellia* (Figure 3). In addition *Hb-hsp*90-1 and *Ce-hsp*90 genes share other two intron positions: the first and fifth from *H. bacteriophora* coincide with the first and second in *C. elegans* (Figure 3).

### Expression Profiles of *Hb*-*hsp*90-1 in Adults and IJs During Parasitic Development

**FIGURE 4:**
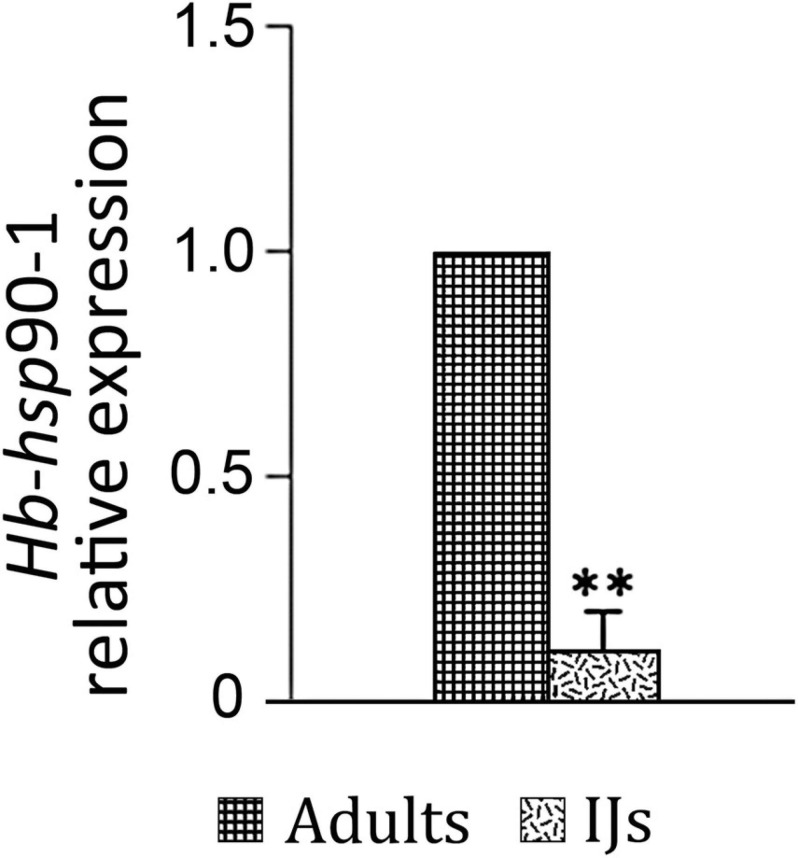
Expression of the *Hb*-*hsp*90-1 in Infective Juveniles (IJs) and adults. Bars indicate standard error of mean data (*n* = 3). (**P < 0.01).

To evaluate the expression of *Hb*-*hsp*90-1 during invasion of *G. mellonella* host (Lepidoptera, Pyralidae), we isolated IJs and adults and determined their expression profiles. As shown in Figure 4, the *Hb*-*hsp*90-1 gene was constitutively expressed in the examined stages, but the *Hb*-*hsp*90-1 expression level was 10-fold higher in adults than in the IJs (Figure 4), thus suggesting that it plays a different role during different life stages.

### Evaluation of the Effects of Lower and Higher Temperatures on Expression Profiles of *Hb*-*hsp*90-1 in Adults and IJs

**FIGURE 5:**
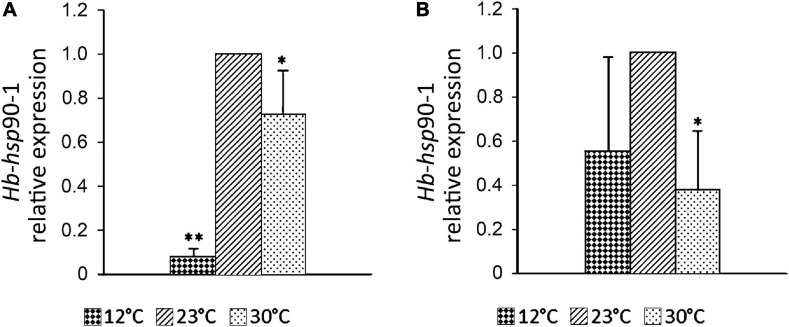
Expression profiles of the *Hb*-*hsp*90-1 of *Heterorhabditis bacteriophora* at 12°C, 23°C, and 30°C in IJs **(A)** and adults **(B)** in aqueous solution. Bars indicate standard error of mean data (*n* = 3). Significant differences (*P < 0.01; **P < 0.05) were found between control and treated nematodes.

In order to explore the effects of temperature levels on gene expression in *H. bacteriophora*, adults and IJs were incubated in aqueous suspension at 12 and 30°C, which are temperatures occurring in Apulia, respectively in winter and summer. Light microscope observations revealed that nematodes had promising levels of heat tolerance. Exposure of IJs to 12°C reduced their relative level of *Hb-hsp*90-1 transcript by 90% (*p* < 0.01) compared to the control at 23°C, while exposure to 30°C caused a 30% reduction (*p* < 0.05) (Figure 5A). Exposure of adults to 12°C reduced the relative level of *Hb-hsp*90-1 transcript by 45%, while exposure to 30°C caused a 62% reduction (*p* < 0.05) (Figure 5B). These data showed the lower and upper temperatures of 12 and 30°C, as initiating heat stress temperatures in *H. bacteriophora*.

### Pathogenicity Analysis at the Lower and Higher Temperatures

Last-instar larvae of *G. mellonella* were infected with *H. bacteriophora* and then incubated at 12, 23, and 30°C for 10 days in order to determine the ability of IJs to be pathogenic. At 23°C, infected *G. mellonella* larvae died between the fourth and sixth day after infection; at 30°C, all infected *G. mellonella* larvae died after the fourth day after infection; at 12°C *G. mellonella* larvae survived or died of natural causes and no EPN reproduction was observed. At 23°C, adults and IJs were collected on the fifth and tenth day, respectively; at 30°C both adults and IJs were collected on the fifth and eighth day, respectively. Our results confirmed that IJ emergence from cadavers is related to temperature, with greater IJ emergence at 30°C than at 23°C. These results showed that the infectivity and reproductive potential of *H. bacteriophora* increased with increasing temperature (30°C), as observed by other authors (Tarasco et al., 2015; Raheel et al., 2017), whereas the low temperature caused the inactivity of the IJs in combination with their symbiotic bacteria.

### Effect of Higher Temperature on *Hb-hsp*90-1 Expression During Parasitic Development

Infected last-instar larvae of *G. mellonella* were incubated at 23 and 30°C for 10 days to evaluate the expression of *Hb-hsp*90-1during invasion of *G. mellonella* and exposure to higher temperature. Adults and IJs were collected as described in the previous section and expression levels of *Hb-hsp*90-1 were measured.

**FIGURE 6:**
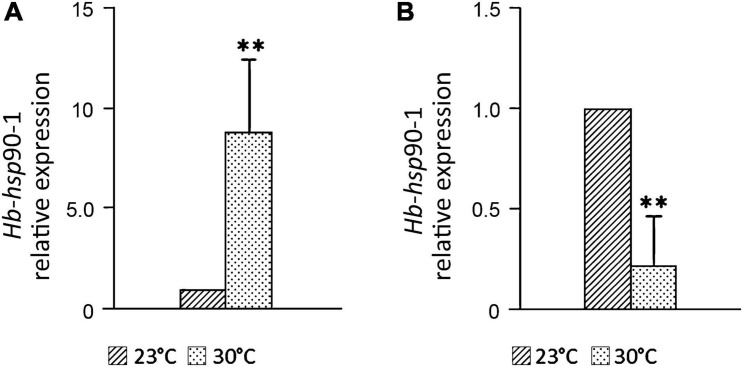
Expression profiles of the *Hb*-*hsp*90-1 of *Heterorhabditis bacteriophora* at 30°C in Infective Juveniles (IJ), **(A)** and adults **(B)** during parasitic development. Bars indicate standard error of mean data (*n* = 3). Significant differences (**P < 0.05) were found between temperature control and higher temperature.

The expression level of *Hb-hsp*90-1 at 30°C in IJs (Figure 6A) increased 8.84 times (*p* < 0.01) compared to the control at 23°C, but decreased by 80% (*p* < 0.01) in adults (Figure 6B). The up-regulation of *Hb-hsp*90-1 in IJs confirmed this gene’s important role for a normal lifespan and protection of protein folding at higher temperatures suggesting a continuous protective mechanism contributing to the tolerance of this strain. On the contrary, *Hb-hsp*90-1 expression in adults decreased in comparison with the control, suggesting that it plays a different role during their development.

### Functional Analysis of dsRNA *Hb*-*hsp*90-1 Under Different Temperature Conditions

**FIGURE 7:**
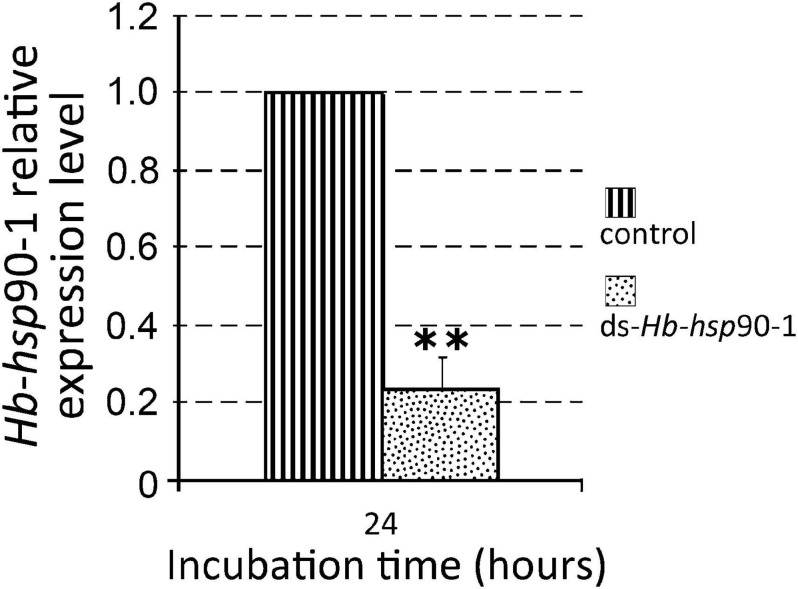
Expression of *Hb*-*hsp*90-1 in Infective Juveniles (IJs) of *Heterorhabditis bacteriophora* treated with dsRNA. Control consisted of untreated IJs for 24 h at 23°C. Significant differences (**P < 0.01) were found between treated and untreated IJs.

**FIGURE 8:**
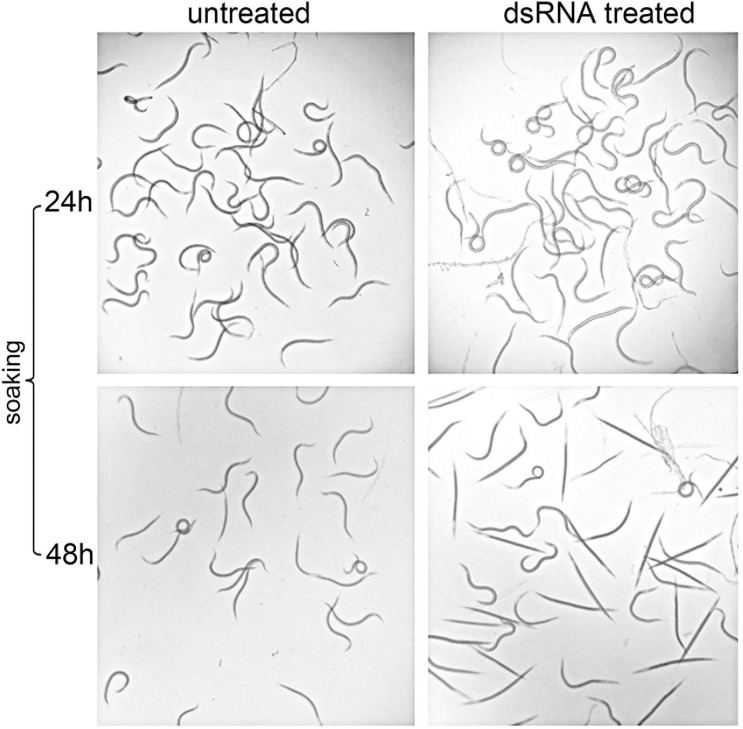
Light microscopy observations of RNAi mediated phenotypes of Infective Juveniles (IJs) of *Heterorhabditis bacteriophora* treated with dsRNA *Hb*-*hsp*90-1 after 24 and 48 h soaking at 23°C.

**FIGURE 9:**
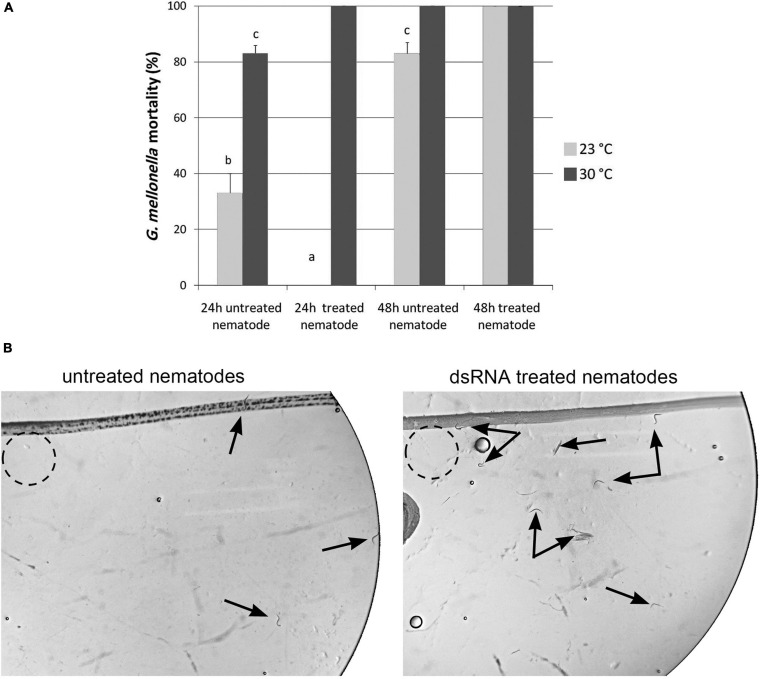
Mean percentage mortality of *Galleria mellonella* larvae after 24 and 48 h following exposure to dsRNA treated Infective Juveniles (IJs) and untreated Infective Juveniles at 23 and 30°C **(A)**. Different letters above bars indicate statistical differences. Untreated and silenced Infective Juveniles (IJs) were transferred on Petri dishes, containing water agar layer, in presence of *Galleria mellonella* larvae to assess nematode attraction **(B)**.

In order to assess the efficiency of the RNAi method, the expression levels of silenced *Hb-hsp*90-1 were determined in IJs and a consistent and statistically significant (*P* < 0.01) 76% reduction was observed between untreated and dsRNA-treated IJs after 48 h of soaking at 23°C (Figure 7). The effect of gene silencing on nematode phenotype was also assessed by light microscope, revealing motile inhibition of silenced *Hb-hsp*90-1 IJs (Figure 8). Untreated and silenced IJs were transferred in Petri dishes, containing a water agar layer, in the presence of unrestrained *G. mellonella* larvae (Figure 9B). After 4 h several untreated nematodes were visible at the release point, while after 24 h, no untreated nematodes were present at the release point. However, treated nematodes after 4 h, were at the release point (data not shown), and after 24 h several treated nematodes were still present at the release point (Figure 9B). After 48 h no silenced IJs were present at the release point (data not shown). These observations seem to suggest that the silencing of *Hb-hsp*90-1 impaired IJs thus reducing the ability to leave the release point and to locate *G. mellonella* compared to untreated IJs. Furthermore, this observation is consistent with the role of Hsp90 for normal lifespan and chemoreception in *C. elegans* (Birnby et al., 2000).

In order to further explore the role of *Hb-hsp*90-1, we investigated the infection and pathogenicity of silenced IJs in last-instar *G. mellonella* larvae at 23°C and 30°C.

After 24 h at 23°C and at 30°C, it was observed that untreated nematodes produced a *G. mellonella* mortality rate of 33 and 83%, respectively. After 24 h at 23 and 30°C, treated IJs produced no *G. mellonella* mortality at 23°C, while produced a larval mortality rate of 100% at 30°C (Figure 9A). After 48 h, treated and untreated nematodes caused high percentage of larvae mortality (>80%) at both 23 and 30°C (Figure 9A). These results clearly confirmed that the *Hb-hsp*90-1 gene is required for recovery process during normal growth and exposure to higher temperatures. Thus IJs are the thermotolerant stage of *H. bacteriophora.*

**FIGURE 10:**
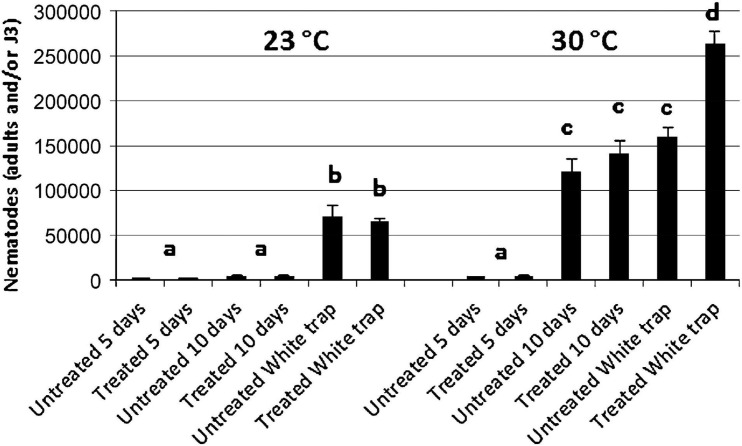
Number of adults and/or Infective Juveniles recovered from *Galleria mellonella* larvae after 5 and 10 days from infection with treated and untreated Infective Juveniles (IJs) at 23 and 30°C. Bars with the same letter have no significant differences (*P* < 0.05).

**FIGURE 11:**
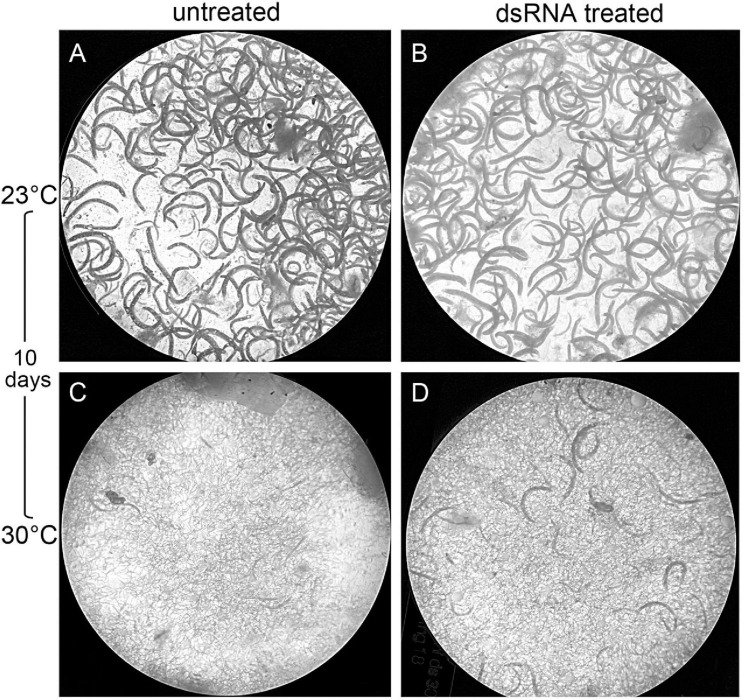
Effects of temperatures on the reproductive potential of treated and untreated *Heterorhabditis bacteriophora* inside *Galleria mellonella* larvae after 10 days infection. **(A)** untreated nematodes at 23°C; **(B)** dsRNA treated nematodes at 23°C; **(C)** untreated nematodes at 30°C; **(D)** dsRNA treated nematodes at 30°C.

The untreated and dsRNA-treated *G. mellonella* were incubated at 23 and 30°C and dissected after 5 and 10 days in order to count adults and IJs. After 5 days at 23°C, the same number of adults were found in infected *G. mellonella* for both the untreated and treated nematodes, while after 10 days, no significant differences in numbers of adults and IJs were observed between untreated and treated nematodes (Figures 10, 11). After 5 days at 30°C, no significant differences were observed for adults and IJs between treated and untreated *G. mellonella*; but after 10 days at 30°C, adults and IJs were counted and no significant differences were observed between treated and untreated (Figures 10, 11). After 10 days at 23°C, no significant differences in numbers of adults and IJs, recovered using White traps, were observed between untreated and treated nematodes, but significantly higher than those recovered from dissected *G. mellonella*. After 10 days at 30°C, the numbers of adults and IJs in White traps were significantly higher (almost double) in *G. mellonella* infected with treated nematodes than those obtained with untreated nematodes (Figures 10, 11). These observations further confirmed that the *Hb-hps*90-1 gene plays a role in the recovery process, increasing the reproduction potential of *H. bacteriophora*, and in protecting it from higher temperature.

## Discussion

Soil is the natural habitat of EPNs and protects them from harmful environmental conditions. Their failure as efficient and effective biocontrol agents may be due to the interaction of different factors affecting their performance, such as ultraviolet radiation, extreme temperatures, and desiccation caused by low moisture levels (Shapiro-Ilan et al., 2006). The aim of this study was to elucidate at the molecular level the adaptability of Apulian *H. bacteriophora*, and also to investigate its development at different temperature levels in order to evaluate the effects of climate change on its host-seeking strategy and efficacy as a biocontrol agent. Since little is known about its molecular response to temperature changes the present study investigated the characterization and function of the *Hb*-*hsp*90-1 gene in *H. bacteriophora* and also examined its role in normal development and recovery process under different temperature conditions, i.e., no stress, low and high temperatures.

To date, little research has focused on the sequence structure and diversity of the *hsp*90 genes in *Heterorhabditis* and *Steinernema* spp. nematodes. The present study reports for the first time the characterization of five isoforms of *Hb-hsp*90 gene (named *Hb-hsp*90-1, *Hb-hsp*90-2, *Hb-hsp*90-3 to 5) in *H. bacteriophora* which differ in sequence diversity and in the presence/absence of introns. Sequence diversity and gene copy number variations (CNV) have been reported in plants, animals and in the nematode model *C. elegans* revealing that these mechanisms are important for genomic variation and usually affect gene families involved in adaptation to stressful or new environments (Maydan et al., 2010; Locke et al., 2015; Zmienko et al., 2016). It has been also reported by Farslow et al. (2015) that these mechanisms also occurred in *C. elegans* populations cultured in laboratory conditions. This finding provided evidence that these changes may be adaptative to laboratory conditions and then fixed. Thus the occurrence of different *hsp*90 DNA fragments in the Apulian *H. bacteriophora*, maintained in laboratory conditions, provide insights that these mechanism could be active in the EPN contributing to a *de novo* genetic novelty for *hsp*90 fragments and so the Apulian *H. bacteriophora* could be more prone to stressful and novel environments.

The intron-rich *Hb-hsp*90-1 isoform was fully characterized showing 99% identities with the annotated sequence (Hba_19207) of *H. bacteriophora* genome present in WormBase Parasite. The deduced protein determined in the present study is shorter than that reported in WormBase Parasite, showing the same length with other nematode Hsp90. *Hb-*Hsp90-1protein revealed the five highly conserved signature sequences and the conserved motif (MEEV) at the C-terminus (Figure 2). Sequence analyses revealed that *Hb-hsp*90-1 gene has eleven introns and 12 exons less than the Hba_19207 sequence showing twelve introns and 13 exons which predicted using bioinformatic programs. Introns are short, like those in the corresponding *hsp*-90 genes of other nematodes thus confirming that introns are under natural selection (Blumenthal and Spieth, 1996; De Luca et al., 2009; Carmel et al., 2012). A cross-species comparison demonstrated that genes involved in stress response, cell proliferation, differentiation and development have short introns and lower intron densities than genes with other functions (Jeffares et al., 2006). Moreover, organisms with short generation times, such as the free-living nematode *C. elegans*, have short introns and a significantly lower genome-wide intron density. This is because they are exposed to rapid environmental changes and so must rapidly respond to changing conditions, thus suggesting that introns may be detrimental to this process (Li et al., 2017). However, the intron density of *Hb-hsp*90-1 is identical to *Bp-hsp*90 of *B. pahangi* and *Mt-hsp*90 of *M. artiellia* and higher than *C. elegans daf*-21, which suggest that parasitic nematode’s introns might contribute to optimal gene expression, generate alternative spliced transcripts and increase the diversity in orthologous genes with identical function. The comparison of intron positions among the four nematode *hsp*90 homologs revealed three introns (Figure 3; 1, 5, and 11 introns) of *Hb-hsp*90-1 in conserved positions compared with *C. elegans daf*-21, and that only the last intron of *Hb-hsp*90-1 was in a conserved position in all four nematodes, suggesting that it is ancestral in nematodes (Rogozin et al., 2005; Henricson et al., 2010). Only two introns (2 and 8) of *Hb-hsp*90-1 were in conserved positions compared with the two parasitic nematodes *M. artiellia* and *B. pahangi*. Introns in conserved positions among orthologous genes are indicative of the functional importance of these introns within the coding region and suggest that they were already present in the equivalent positions of the corresponding ancestral gene. Thus, we can speculate that higher number of introns in *Hb-hsp*90-1 may affect the expression of the gene at several levels including stability and translation efficiency and adaptation to environmental changes. These findings seem to confirm that the number and the positions of introns in homologous *hsp*90 genes have changed during evolution providing a source of information about the evolutionary past (Carmel et al., 2007; Chorev and Carmel, 2012). The occurrence of the three intronless isoforms, *Hb-hsp*90-3 to 5 in the Italian strain of *H. bacteriophora*, also present in individual nematodes, suggests that they may be retropseudogenes or paralogous genes arising by gene duplication events with different sequence and gene structures leading to organismal evolution and acquisition of new functions (Katju and Lynch, 2003; Baskaran and Rödelsperger, 2015). A recent report documents two isoforms of the *hsp*90 gene in the plant parasitic nematode *Pratylenchus penetrans*, these differ in their nucleotide sequence and genomic structure (the presence of one or two introns) and are the result of independent duplication events (Pantzartzi et al., 2013; Fanelli et al., 2018). The occurrence of the *Hb-hsp*90-2 retrogene, which is 99% identical to *Hb-hsp*90-1 cDNA, is of great interest and emerged via reverse transcription of the corresponding mRNA, then randomly inserted into the genome. The partial duplication of *Hb-hsp*90-2 may be a non-functional gene fragment, and thus it can be speculated that this is an intronless intermediate, which then might accumulate new introns and new functions or may subsequently be deleted from the genome (Bai et al., 2007; Milanowski et al., 2016). *Hb-hsp*90-2 may be a young retropseudogene that is still recognizable because of its high sequence identity with *Hb-hsp*90-1. The *in silico* and phylogenetic analyses using all *Hb-hsp*90 sequences determined in this study show that all *H. bacteriophora* sequences group in different clusters, supporting the existence of different *Hb-hsp*90 isoforms (Figure 1) as shown in *P. penetrans* and other Eukaryotes. It has been reported in humans that the rate of retention of *hsp*90 duplicates is high because this gene family is involved in stress response (Pantzartzi et al., 2013). Our analyses support the existence of multiple genes in *H. bacteriophora* and the retention of duplicated genes could be correlated to different environmental stimuli upon their transfer from soil to insect hosts. Therefore, it will be necessary to prove whether these are expressed or defective copies of functional genes.

EPNs have adapted to develop and function within temperature ranges typical for their habitat, and so environmental characterization of EPNs can help in the selection of the strains most useful to control pests (Susurluk and Ehlers, 2008; Mukuka et al., 2010b; Shapiro-Ilan et al., 2014; Ulu and Susurluk, 2014). This process of heat acclimation increases thermotolerance and the duration of heat endurance (Treinin et al., 2003). Most *H. bacteriophora* strains have been isolated in sandy soil and near the sea, thus resulting a warm-adapted nematode. The current study demonstrates that the indigenous Apulian *H. bacteriophora* strain is well adapted to warm temperatures, since it can infect *G. mellonella* between 23 and 30°C. Furthermore, its reproduction was higher at 30°C, when it proved capable for killing its host more rapidly than at the control temperature. Conversely, the Apulian *H. bacteriophora* population was unable to reproduce at 12°C in *G. mellonella* indicating that this strain is not cold tolerant as IJs are already arrested stages adapted to withstand the unfavorable environmental conditions outside the host (De Luca et al., 2009). These results confirm the direct effects of the temperature on *H. bacteriophora* infectivity and pathogenicity as also observed by Castelletto et al. (2014).

Little is known about the molecular pathways or genes involved in the *H. bacteriophora* recovery process and signaling in response to temperature changes. Recent data showed that of heat shock proteins induced during the recovery process of *H. bacteriophora* IJs, *Hb-hsp*90-1 gene was up-regulated (Moshayov et al., 2013), confirming that IJs experience heat shock as part of their life cycle when they transfer from soil to host insects. It has been demonstrated in three plant parasitic nematodes, *M. artiellia*, *P. vulnus*, and *B. xylophilus*, and in the free-living nematode *C. elegans*, that the *hsp*-90 gene is involved in normal larval development, dauer formation regulation, chemosensation, proteostasis and response to temperature changes (De Luca et al., 2009; Wang et al., 2012; Fanelli et al., 2019). Chemotaxis in EPNs plays an important role in their host-seeking behavior, temperature changes can cause variability in host selection and can negatively affect EPN efficacy as biocontrol agents (Chaisson and Hallem, 2012; Lee et al., 2016). It has been reported that several heat shock proteins are induced in response to temperature changes enhancing tolerance of *Heterorhabditis* species to higher temperature (Mukuka et al., 2010a, b). Thus, we evaluated how *Hb-hsp*90-1 expression changed during normal growth and in response to temperature changes. Our results revealed that *Hb-hsp*90-1 is constitutively expressed in the studied developmental stages. Moreover, the level of *Hb-hsp*90-1 transcripts in adults is 10-fold higher than in IJs, thus confirming that *H. bacteriophora* IJs are arrested metabolic stages and the increase of *Hb-hsp*90-1 level during the recovery process reflected the transition from the arrested stages to the parasitic stages (Figure 4). In addition, the higher level of *Hb-hsp*90-1 in adults indicates that this gene is differentially regulated during normal growth. Exposure of *H. bacteriophora* IJs and adults in aqueous medium to different temperatures (12, 23, and 30°C) for 6 h, revealed that *Hb-hsp*90-1expression was down-regulated in both stages at 12 and 30°C, compared with 23°C, confirming the direct influence of temperature on these stages of *H. bacteriophora* (Figure 5). To validate the relationships between temperature increase, parasitic behavior and reproduction, transcript levels of *Hb-hsp*90-1 were determined in IJs and adults recovered from *G. mellonella* at 23 and 30°C after 10 days of incubation (Figure 6). *Hb-hsp*90-1 expression was up-regulated in IJs (Figure 6A), confirming that *Hb-hsp*90-1 is induced during the recovery process and thus involved in parasitic behavior within a restricted temperature range (23–30°C); an 80% reduction in the *Hb-hsp*90-1 level was actually observed in adults (Figure 6B). These results clearly demonstrate that IJs are the temperature tolerant stages of *H. bacteriophora* suggesting that *Hb-hsp*90-1 may act as chaperone for those molecules involved in temperature increase along with those involved in chemosensation and in interaction with *G. mellonella* hosts. On the contrary, adults reduced *Hb-hsp*90-1 expression to cope with adverse environmental conditions suggesting that adults are not thermotolerant stages. Thus, our results suggest that *Hb-hsp*90-1 could be also a part of the olfactory response in *H. bacteriophora* as found in other parasitic and free-living nematodes suggesting that this gene can optimize the adaptation of IJs to host life stages during seasonal temperature changes as also observed by others authors (Chaisson and Hallem, 2012; Lee et al., 2016).

To further confirm the function of *Hb-hsp*90-1 during normal lifespan and temperature increase, the gene was knocked down using RNAi as described in literature (Fanelli et al., 2005; Ciche and Sternberg, 2007; Lourenco-Tessutti et al., 2015) and the effect of gene silencing was followed in the IJs. Silenced IJs showed motile inhibition and behavioral aberration when observed using light microscopy (Figure 8), together with a 76% reduction in the *Hb-hsp*90-1 transcription level (Figure 7). The comparison of the behavior of treated and untreated IJs, laid at a specific release point within the agar plates in presence of *G. mellonella* larvae, revealed that both treated and untreated IJs were able to move within the release point, but whereas untreated IJs left the release point after 4 h, treated IJs were able to leave it after 24 h (Figure 9B). This finding prompted us to infer that the *Hb-hsp*90-1 silencing somehow impaired the motility in treated IJs. These results clearly demonstrated that the silencing effect is transient and not detectable after 24 h, highlighting the key role of *Hb-hsp*90-1 during the normal lifespan and it could be also involved in chemosensation in EPNs.

It was then tested whether temperature changes can alter the parasitic behavior of *H. bacteriophora*, using, untreated and silenced IJs, to infect *G. mellonella* cultured at 23 and 30°C. After 24 h, the mortality percentage of *G. mellonella* infected with untreated IJs at 23°C was lower than that of *G. mellonella* infected with untreated IJs at 30°C (Figure 9A). After 24 h, *G. mellonella* infected with silenced IJs showed no mortality at 23°C while those infected with silenced IJs at 30°C showed 100% mortality (Figure 9A). After 48 h, the percentage of larva mortality infected with the untreated IJs increased both at 23 and 30°C, but *G. mellonella* mortality was still higher at 30°C than at 23°C. After 48 h, the mortality percentage of *G. mellonella* infected with silenced IJs at both 23 and 30°C was higher (Figure 9A). These results show that RNAi on IJs of *H. bacteriophora* works well and it is effective at 23°C. Conversely, the dsRNA is no longer stable at 30°C and thus nematodes can reproduce more rapidly. All together these observations confirm that *Hb-hsp*90-1 is required for host location and the recovery process. It appears that a temperature of 30°C did not alter the recovery process of *H. bacteriophora* when infected *G. mellonella* larvae were cultured under constant temperature conditions. These observations clearly demonstrate that IJs are the thermotolerant stages of Apulian *H. bacteriophora*.

The dissection of *G. mellonella* infected with untreated and treated IJs to 23°C for 5 days revealed the same number of nematodes; after 10 days there were no significant differences between infected *G. mellonella* with untreated and treated IJs in the number of nematodes (Figures 10, 11). The dissection of *G. mellonella* infected with untreated and treated IJs to 30°C for 5 days revealed no significant differences in the number of nematodes between infected *G. mellonella* with untreated and treated IJs; after 10 days at 30°C no significant differences in the number of nematodes between infected *G. mellonella* with untreated and treated IJs (Figures 10, 11), but it was higher than that after 5 days. It is noteworthy that, after 10 days at 30°C, the numbers of adults and IJs in the White traps containing *G. mellonella* infected with silenced nematodes were significantly higher than those found in *G. mellonella* with untreated nematodes (Figures 10, 11). Thus, we can speculate that the continuous and higher expression of *Hb-hsp*90-1 can contribute to the survival of nematodes exposed to a higher temperature for a long time allowing *H. bacteriophora* IJs to acclimate, to modulate the recovery process and to enable them to target seasonal hosts or host life stages. In conclusion, our data demonstrate, for the first time, that *Hb-hsp*90-1 plays an important role in the recovery process for normal development and during temperature increases. Furthermore, these results seem to suggest that the expression level of *Hb-hsp*90-1 can contribute to the strong correlation between IJs lifespan and heat tolerance in *H. bacteriophora*, thus contributing to generate adapted and non-adapted strains. The occurrence of duplicated isoforms of *Hb-hsp*90-1 gene suggests that these may contribute to increasing the efficacy of the native strain of *H. bacteriophora* for insect control in the field. Finally, this study provides evidence that silencing by soaking can be used with success in *H. bacteriophora*, but after 24 h it is transient and unstable thus revealing that soaking technique can be used for identification of gene function and expression in EPNs.

## Data Availability Statement

The data presented in the study are deposited in the GenBank repository, accession numbers: LR890107–LR890114 for *Hb-hsp*90-1 sequences; LR890106 for mRNA *Hb-hsp*90-1; LR890105 for *Hb-hsp*90-2; LR890115–LR890117 for *Hb-hsp*90-3; LR890118–LR890120 for *Hb-hsp*90-3; LR890121-LR890123 for *Hb-hsp*90-5.

## Author Contributions

EF and FD conceived and designed the experiments and oversaw the manuscript preparation. EF performed the laboratory experiments and contributed to writing the results. AT counted nematodes and performed the microscope observations. ET performed the nematode cultures and stage separations. FD analyzed the data analysis and wrote the manuscript, which was read and approved by all authors.

## Conflict of Interest

The authors declare that the research was conducted in the absence of any commercial or financial relationships that could be construed as a potential conflict of interest.
